# HeteroGrad-DT: Gradient Flow Transformer for heterogeneous multi-task offline reinforcement learning

**DOI:** 10.1371/journal.pone.0362298

**Published:** 2026-11-11

**Authors:** Ye Wang, Donghui Ma, Jiang Li, Lihong Guo

**Affiliations:** 1 Changchun Institute of Optics, Fine Mechanics and Physics, Chinese Academy of Sciences, Changchun, Jilin, China; 2 University of Chinese Academy of Sciences, Beijing, China; Tsinghua University, CHINA

## Abstract

Offline reinforcement learning enables policy learning from static datasets without online exploration, making it particularly suitable for robotic control under safety and data constraints. Decision Transformer recasts offline reinforcement learning as conditional sequence modeling. Extending it to heterogeneous multi-task settings is challenging because task representations and dynamics differ, while joint training can suffer from destructive gradient interference. To address these challenges, we present HeteroGrad-DT, an integrated framework that employs task-specific tokenizers and decoupled prediction heads to retain task-native input and output dimensionality at the model interfaces while learning a shared sequence backbone. It further introduces a Gradient Flow Transformer that periodically coordinates cross-task updates using fixed-dimensional task statistics, compact summaries of shared-backbone gradients accumulated within the current synchronization interval, and an alignment-based coordination objective, rather than relying only on stepwise projection or static regularization. Experiments on the DeepMind Control Suite with up to 30 tasks show that HeteroGrad-DT improves average normalized return over representative multi-task methods. In the 30-task setting, it achieves a 32.3% relative improvement over standard training with a shared backbone and an 8.6% gain over a representative gradient-conflict mitigation baseline using the same task-aware interfaces. Ablation, mechanism, and sensitivity analyses further support complementary benefits from task-aware interfaces and gradient coordination in the evaluated settings.

## Introduction

Offline reinforcement learning (offline RL) has emerged as a practical paradigm for learning control policies from static datasets without requiring online interaction. This paradigm is particularly valuable in robotics, autonomous systems, and industrial control, where online exploration may be unsafe, expensive, or infeasible. In single-task settings, offline RL methods have made substantial progress by addressing distribution shift and value overestimation through behavior modeling, behavior regularization, and conservative value estimation [1–4]. These methods have significantly improved the reliability of offline policy optimization. However, they often rely on explicit value learning, carefully designed regularization, or explicit restrictions around the support of the offline dataset, which can limit modeling flexibility and make it difficult to build a single decision-making model across diverse control behaviors.

More recently, Decision Transformer (DT) [5] has offered a compelling alternative by reformulating offline RL as conditional sequence modeling. Instead of learning policies through explicit Bellman backup, DT models trajectories autoregressively and conditions decisions on return-to-go (RTG), states, and past actions. This sequence-modeling view avoids the instability of value-based learning with bootstrapped targets, naturally captures long-horizon dependencies, and provides a flexible architecture for learning directly from offline trajectories. Owing to these advantages, DT has become an appealing foundation for adapting offline RL beyond single-task learning.

Despite this progress, many practical control scenarios require policies to handle multiple related tasks rather than a single isolated behavior. This motivates the extension of DT-style sequence modeling to multi-task settings, especially when tasks differ in state representations, action spaces, and dynamics. Recent studies have begun to adapt DT-style frameworks to multi-task settings through shared backbones, task prompts, and lightweight task-conditioning mechanisms [6–8]. These efforts show that sequence-based offline decision models can support cross-task knowledge sharing within a common architecture. However, existing multi-task DT methods are typically most effective when tasks are relatively aligned and can be incorporated into a shared model with limited adaptation. In diverse control domains, tasks may differ substantially in state dimensions, action spaces, semantic structure, and underlying dynamics. Directly sharing a backbone or adding task prompts is therefore often insufficient to resolve the structural and optimization conflicts that arise during joint training. Two closely related obstacles are particularly important.

**Challenge 1: Heterogeneous State-Action Representations.** Heterogeneous tasks are often forced into a shared interface to enable joint training, yet naive alignment can overlook semantic differences across dimensions. A common workaround is zero-padding, which forces shorter state vectors to match longer ones by appending zeros and thereby introduces artificial dimensions with no semantic content. This causes the shared model to treat structurally incomparable features as if they were aligned, which can blur task-specific representations and entangle task identity with task-dependent padding patterns. As a result, knowledge sharing may degrade when the shared interface must resolve task-dependent dimensional mismatch within nominally shared feature coordinates. Prompt conditioning or training with a shared backbone does not directly remove this representation mismatch because artificial padded dimensions still enter the supervised sequence-modeling objective.

**Challenge 2: Cross-Task Gradient Interference in Shared Optimization.** Even when heterogeneous tasks are mapped into a common backbone, joint training remains difficult because gradients from different tasks may induce conflicting update directions. This issue remains important in offline multi-task learning, where all tasks compete for a shared parameter space throughout joint training. Existing strategies for mitigating gradient conflict, including GradNorm, MGDA, PCGrad, CAGrad, and Nash-MTL [9–13], mainly operate through loss reweighting or local correction of incompatible gradients. These methods can reduce instantaneous conflicts, but the proposed Gradient Flow Transformer (GFT) instead coordinates task updates using optimization information aggregated within the current synchronization interval.

To address these challenges, we present HeteroGrad-DT, an integrated extension of Decision Transformer for heterogeneous multi-task offline reinforcement learning. Heterogeneity-Aware Prompt-DT (HAP) reduces state-action interface mismatch through task-specific tokenizers and prediction heads, while GFT coordinates conflicting optimization signals through periodic learned fusion. Our main contributions are summarized as follows:

- **Task-Specific Tokenization and Decoupled Prediction.** We introduce HAP, which uses task-specific tokenizers and decoupled prediction heads around a shared sequence backbone. This design avoids explicit zero-padding across heterogeneous task spaces while retaining task-native input and output dimensionality at the model interfaces. It allows the shared Transformer to model temporal regularities across tasks without forcing all tasks into a fully shared input and output space.
- **Gradient Flow Transformer.** We introduce GFT, which periodically coordinates cross-task updates using accumulated optimization signals. Instead of only correcting instantaneous gradient conflicts at each step, GFT learns adaptive fusion weights from Task Statistics and Gradient Digest features. The resulting update encourages higher average alignment across tasks and reduces harmful interference during heterogeneous multi-task training.
- **Empirical Evaluation.** We evaluate HeteroGrad-DT on the DeepMind Control Suite with up to 30 tasks and report five-seed statistics with confidence intervals. The experiments include comparisons with representative multi-task methods, component-removal ablation studies, HAP compatibility across backbones, action-prediction analysis, gradient-conflict analysis, GFT hyperparameter sensitivity, and matched training-cost measurements.

The two challenges motivating the method are summarized in Fig 1.

**Fig 1:**
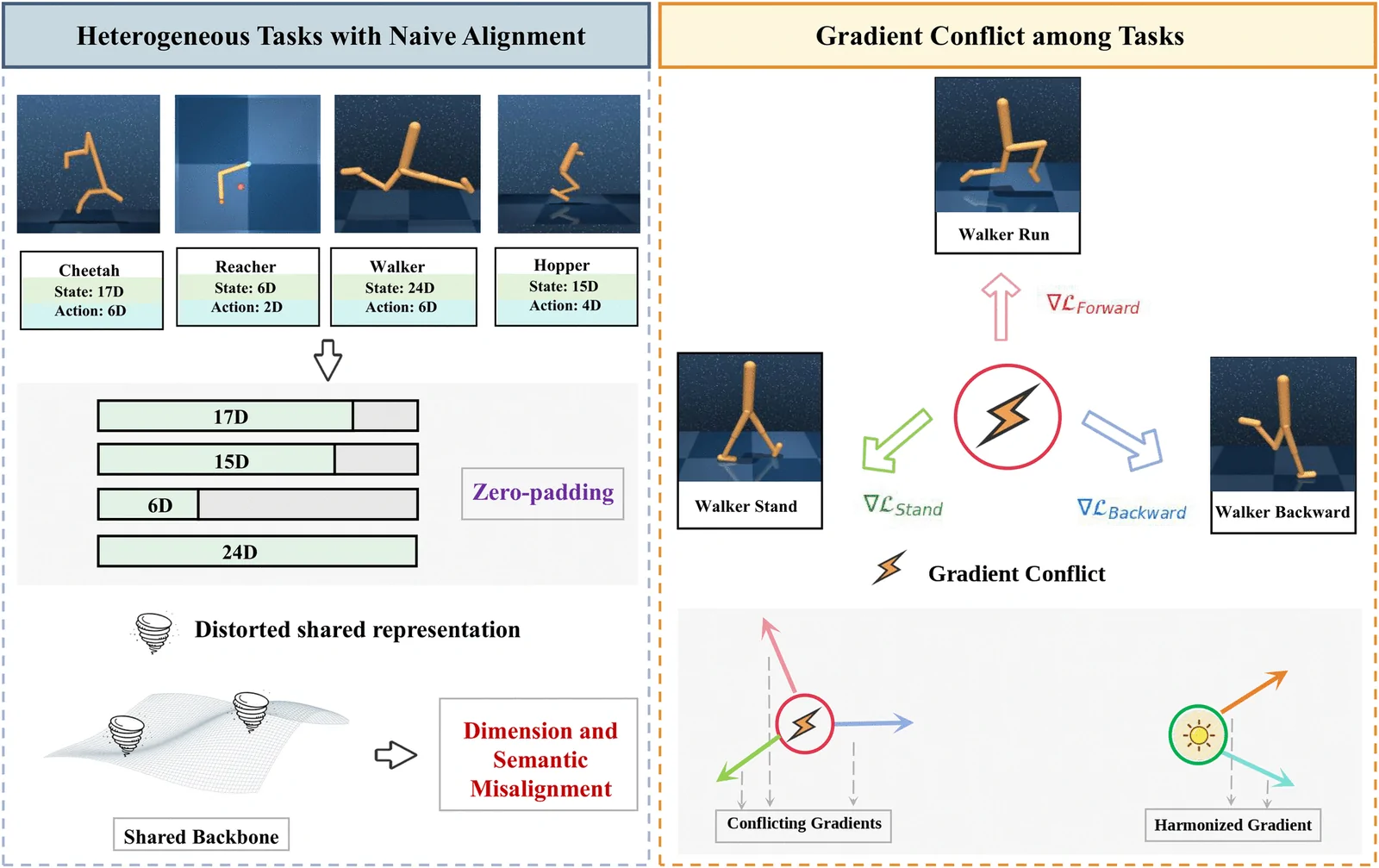
Challenges in offline multi-task reinforcement learning. **Left**: Under heterogeneous tasks with naive alignment, differences in state-action dimensions and feature semantics are mapped into a shared interface, for example through zero-padding, which can distort the shared representation and lead to dimensional and semantic misalignment in the shared backbone. **Right**: Conflicting gradients among tasks produce incompatible update directions during joint training; the harmonized direction illustrates the intended role of cross-task gradient coordination.

## Related work

Reinforcement learning has also been applied to adaptive decision-making in engineering systems, including sensor configuration and measurement selection [14,15]. Within offline reinforcement learning, existing methods have mainly advanced along two directions. A first group of methods aims to keep the learned policy close to the behavior distribution represented in the offline dataset, as in methods based on behavior modeling or policy regularization such as BCQ, BEAR, and BRAC [2,16,17]. A second group improves robustness through conservative value estimation, represented by methods such as CQL and IQL [3,4]. Beyond explicit value-based formulations, recent work has shown that offline control can also be approached through sequence or generative modeling. Representative examples include Decision Transformer, Trajectory Transformer, and RvS [5,18,19], together with extensions such as Q-learning Decision Transformer, Generalized Decision Transformer, Constrained Decision Transformer, Graph Decision Transformer, and Diffuser [20–24].

At a broader scale, transformer-based and generalist agents have demonstrated the scalability of sequence modeling on diverse data. Examples include Gato, RT-1, and Multi-Game Decision Transformers [8,25,26]. In parallel, multi-task reinforcement learning has explored shared-policy learning through transfer, distillation, and modularization, as in Actor-Mimic, Distral, and Soft Modularization [27–29]. These studies highlight the promise of shared architectures, but they also underline a recurring difficulty. As task diversity grows, representation mismatch and optimization interference become increasingly difficult to manage within a single model.

A complementary line of research studies interference in multi-task optimization. Representative approaches include GradNorm, MGDA, PCGrad, CAGrad, and Nash-MTL [9–13], which rebalance losses or manipulate gradients to reduce destructive interactions among tasks. These approaches are mainly studied in generic multi-task learning rather than in heterogeneous multi-task offline sequence modeling, where representation alignment and optimization coordination must be handled together.

More recently, multi-task offline reinforcement learning has adopted sequence-modeling and generative paradigms more directly. Existing methods include shared sequence modeling across diverse tasks, represented by Multi-Game Decision Transformers [8]; prompt conditioning, represented by Prompt-DT [6]; parameter-space masking, represented by HarmoDT [7]; and diffusion-based multi-task planning, represented by MTDiff [30].

These methods demonstrate the promise of shared sequence models, but they primarily emphasize task conditioning, parameter sparsification, or generative planning. HeteroGrad-DT instead focuses on the joint treatment of heterogeneous input/output interfaces and cross-task optimization coordination.

## Materials and methods

This section presents HeteroGrad-DT. An overview of the architecture is shown in Fig 2, and the complete training procedure is summarized in Algorithm 1. We first describe HAP and then introduce GFT.

**Fig 2:**
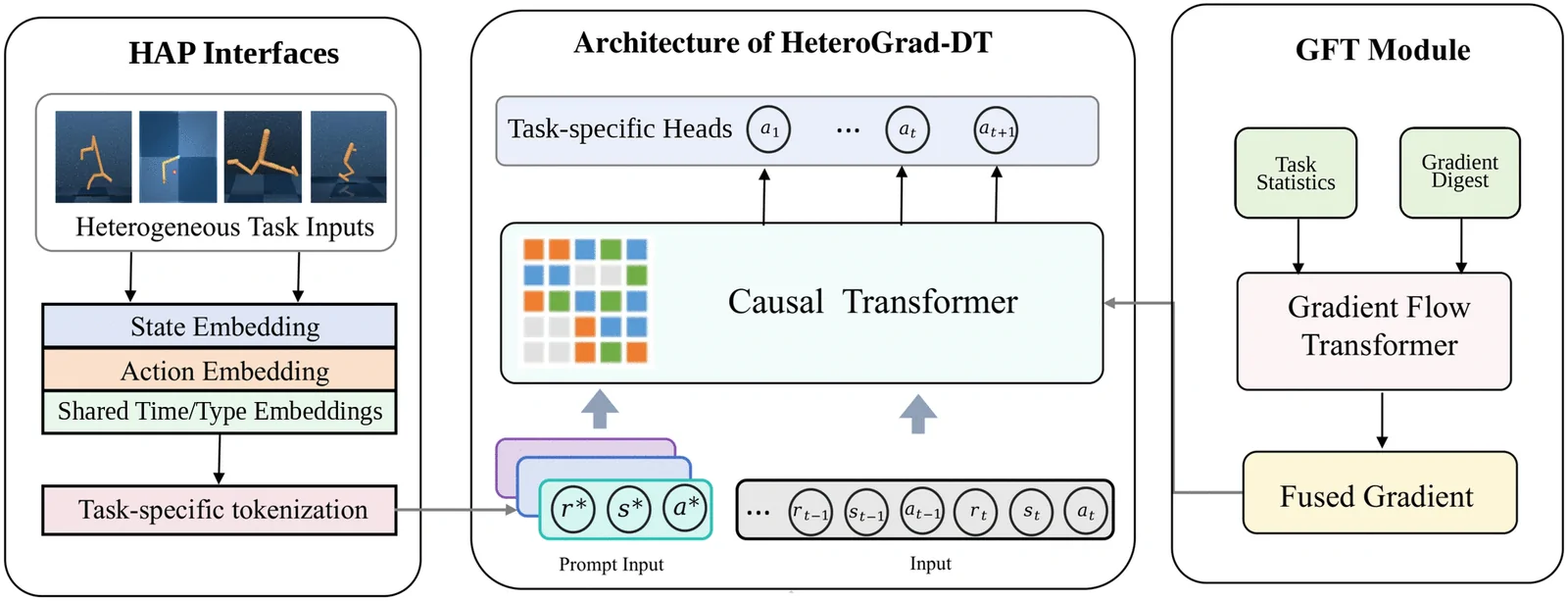
Overall architecture of HeteroGrad-DT. HAP denotes the task-specific state/action tokenizers and task-specific prediction heads surrounding the shared causal Transformer backbone; the scalar RTG embedding and temporal/type embeddings remain shared across tasks. The GFT module uses Task Statistics and Gradient Digest features to produce adaptive fusion weights, which are then applied to interval-mean task gradients to form the fused gradient used to update the shared Transformer.

### Heterogeneity-aware prompt-DT

Real-world control tasks exhibit substantial heterogeneity in observation dimensions and action constraints, which presents a practical challenge for multi-task learning. To bridge disparate task spaces into a common latent representation, we define Heterogeneity-Aware Prompt-DT (HAP) as the task-specific input/output interface composed of task-specific state/action tokenizers and task-specific prediction heads. The shared causal Transformer is treated as a replaceable backbone rather than as part of HAP.

#### Shared prompt-DT backbone.

Prompt-DT utilizes trajectory prompts composed of state, action, and return-to-go tuples. These prompts provide direct task guidance and are especially useful when a short demonstration is available. Formally, elements belonging to the trajectory prompt are marked with a superscript *. The prompt window is much shorter than the full task horizon and provides task context rather than a complete demonstration for direct imitation.

During offline training, for each task at timestep *t*, the model input τ is constructed by combining a $K_{p}$-step trajectory prompt $\tau^{*}$ with the most recent *K*-step history $\tau^{h}$:


$$\begin{array}{c} \tau =\left[\tau^{*};\tau^{h}\right]=(\underset{\text{Prompt }\tau^{*}}{\underbrace{R_{1}^{*},s_{1}^{*},a_{1}^{*},...,R_{K_{p}}^{*},s_{K_{p}}^{*},a_{K_{p}}^{*}}},\underset{\text{History }\tau^{h}}{\underbrace{R_{t-K+1},s_{t-K+1},a_{t-K+1},...,R_{t},s_{t}}}). \end{array}$$ (1)

Here, $K_{p}$ and *K* denote the prompt length and recent-history context length, respectively. Following the standard Prompt-DT construction, RTG is retained as a scalar input modality in both the prompt and the recent history. It is mapped to the shared token dimension by a linear return embedding and combined with the same temporal indexing used by the state and action tokens:


$$\begin{array}{c} e_{R,t}=W_{R}R_{t}+e_{type}^{R}+e_{time}^{t},\;W_{R}\in {\mathbb{R}}^{d\times 1}. \end{array}$$ (2)

The prompt and history therefore follow the same RTG–state–action ordering. HAP changes the task-dependent state and action interfaces and the task-specific action heads; the scalar return embedding and temporal embedding remain shared across tasks.

The prediction head associated with the current state token $s_{t}$ predicts the corresponding action $a_{t}$. For continuous action spaces, the model is trained using the mean-squared error over a batch of size *B*:


$$\begin{array}{c} ℒ=\frac{1}{B}\sum_{b=1}^{B}{\left\|{\hat{a}}_{t}^{\left(b\right)}-a_{t}^{\left(b\right)}\right\|}_{2}^{2}. \end{array}$$ (3)

#### Task-specific tokenizers.

To interface the shared backbone with heterogeneous tasks, we introduce Task-Specific Tokenizers. These modules act as learnable interface layers that resolve structural mismatches between task-native inputs and the shared latent space. Consider task *i* with observation dimension $d_{s}^{\left(i\right)}$ and action dimension $d_{a}^{\left(i\right)}$. To map these inputs into the shared backbone dimension *d*, we use distinct linear projections parameterized by $W_{s}^{i}\in {\mathbb{R}}^{d\times d_{s}^{\left(i\right)}}$ and $W_{a}^{i}\in {\mathbb{R}}^{d\times d_{a}^{\left(i\right)}}$.

The tokenizer combines projected content features with Type Embeddings, which distinguish modalities, and Time Embeddings, which encode temporal position. The resulting state and action tokens are


$$\begin{array}{cc} e_{s,t} & =W_{s}^{i}s_{t}+e_{type}^{s}+e_{time}^{t}, \end{array}$$ (4)

$$\begin{array}{cc} e_{a,t} & =W_{a}^{i}a_{t}+e_{type}^{a}+e_{time}^{t}. \end{array}$$ (5)

This design converts diverse input streams into *d*-dimensional tokens. By handling task-dependent input dimensionality in the tokenizers, the shared backbone can focus on temporal patterns shared across tasks rather than fitting incompatible input dimensions.

#### Independent prediction heads.

To map the shared representations back to task-native action spaces, HAP uses Independent Prediction Heads. This decoupled design avoids forcing tasks with different action dimensions and constraints to compete within a single output layer.

We construct a dictionary of task-specific heads $ℋ=\{h_{1},...,h_{N}\}$. For task *i*, the shared backbone produces a latent vector $z_{s,t}$, which is routed exclusively to $h_{i}$:


$$\begin{array}{c} {\hat{a}}_{t}=h_{i}(z_{s,t})={MLP}_{i}(z_{s,t}). \end{array}$$ (6)

By isolating the final regression layers, structural heterogeneity in the action spaces is handled locally, while the shared backbone learns temporal representations shared across tasks.

### Gradient flow transformer

Let θ denote the shared causal Transformer parameters and ϕ the trainable GFT parameters (descriptor projection, Transformer encoder, and fusion head); task-specific tokenizer/head parameters are updated by the ordinary task-wise optimizer. GFT operates on compact interval-level gradient summaries rather than full gradient trajectories.

#### Optimization state descriptor.

For each task *i*, GFT receives a fixed-dimensional Optimization State Descriptor $f_{i}$ that combines Task Statistics with a Gradient Digest. Let ${𝒟}_{i}$ denote the pre-collected trajectories after the same task-wise state and action preprocessing used by HAP. We summarize the dataset by six scalars:


$$\begin{array}{c} f_{stat}^{\left(i\right)}=\left[d_{s}^{\left(i\right)},d_{a}^{\left(i\right)},\mu_{s}^{\left(i\right)},\sigma_{s}^{\left(i\right)},\mu_{a}^{\left(i\right)},\sigma_{a}^{\left(i\right)}\right], \end{array}$$ (7)

where $\mu_{s}^{\left(i\right)}$ and $\mu_{a}^{\left(i\right)}$ denote means, while $\sigma_{s}^{\left(i\right)}$ and $\sigma_{a}^{\left(i\right)}$ denote standard deviations computed over all valid state or action components and timesteps in ${𝒟}_{i}$. Using scalar aggregate statistics keeps the descriptor length independent of the native state and action dimensions. The six features are standardized across the current task set before the learned descriptor projection.

During one synchronization interval, let ${ℬ}_{i}=\{q_{i}^{\left(1\right)},...,q_{i}^{\left(m_{i}\right)}\}$ contain the shared-backbone gradients recorded at the local updates of task *i*, where $m_{i}=|{ℬ}_{i}|$ denotes the number of gradient records collected for task *i* within the current synchronization interval. The accumulated task gradient is defined as the interval mean


$$\begin{array}{c} g_{i}=\frac{1}{m_{i}}\sum_{k=1}^{m_{i}}q_{i}^{\left(k\right)}. \end{array}$$ (8)

This averaging prevents the descriptor and fused update from depending on small differences in how often a task is sampled within the interval. For the Gradient Digest, we use the coordinate-wise mean and variance of the interval-mean gradient $g_{i}$; thus, $(\sigma_{g}^{\left(i\right)}{)}^{2}$ denotes the variance rather than the standard deviation. For a shared backbone with *p* gradient coordinates, the Gradient Digest is


$$\begin{array}{c} f_{grad}^{\left(i\right)}=\left[\mu_{g}^{\left(i\right)},{\left(\sigma_{g}^{\left(i\right)}\right)}^{2}\right],\;\mu_{g}^{\left(i\right)}=\frac{1}{p}\sum_{\ell =1}^{p}g_{i,\ell },\;{\left(\sigma_{g}^{\left(i\right)}\right)}^{2}=\frac{1}{p}\sum_{\ell =1}^{p}{\left(g_{i,\ell }-\mu_{g}^{\left(i\right)}\right)}^{2}. \end{array}$$ (9)

The final descriptor is


$$\begin{array}{c} f_{i}=\left[f_{stat}^{\left(i\right)}∥f_{grad}^{\left(i\right)}\right]. \end{array}$$ (10)

#### Attention-driven gradient fusion.

The GFT module models relations among task descriptors to generate adaptive fusion weights. The descriptors $F=\{f_{1},...,f_{N}\}$ are projected to the GFT hidden dimension and processed with a Transformer encoder. To preserve permutation equivariance across arbitrary task orderings, the GFT encoder omits task-order positional embeddings. Under this construction, permuting the task descriptors permutes the corresponding output tokens and weights in the same way, while leaving the resulting weighted sum unchanged.

Let $f_{i}^{\;\prime }$ denote the output token for task *i* from the final GFT Transformer layer. A linear fusion head followed by Softmax yields $\alpha =[\alpha_{1},...,\alpha_{N}]$:


$$\begin{array}{c} \alpha_{i}=\frac{\exp\;\left(Linear(f_{i}^{\;\prime })\right)}{\sum_{j=1}^{N}\exp\;\left(Linear(f_{j}^{\;\prime })\right)}. \end{array}$$ (11)

The fused gradient used by the periodic coordination branch is


$$\begin{array}{c} \tilde{g}=\sum_{i=1}^{N}\alpha_{i}g_{i}. \end{array}$$ (12)

The attention layers compare the current task descriptors jointly, whereas the objective below evaluates the agreement between each task gradient and the final fused direction. The mechanism analysis below therefore reports both pairwise conflict and alignment with the final update direction.

#### Shared progress maximization objective.

The fusion policy is trained with the Shared Progress Maximization (SPM) objective. We use the negative average cosine agreement between $\tilde{g}(\phi )$ and the interval-mean task gradients $g_{i}$ as a first-order coordination surrogate:


$$\begin{array}{c} {ℒ}_{SPM}(\phi )=-\frac{1}{N}\sum_{i=1}^{N}\frac{g_{i}^{⊤}\tilde{g}(\phi )}{\left(\|g_{i}{\|}_{2}+\epsilon \right)\left(\|\tilde{g}(\phi ){\|}_{2}+\epsilon \right)}, \end{array}$$ (13)

where ϵ>0 prevents numerical instability when a gradient norm is close to zero. The negative average cosine agreement provides a tractable first-order coordination surrogate that favors a shared direction with higher mean agreement across the tasks observed during the current synchronization interval.

Training alternates between ordinary HAP updates and periodic GFT synchronization. At a local iteration, a task and batch are sampled, the task-specific tokenizer and prediction head are selected, and the ordinary HAP optimizer updates the model using the task loss. The shared-backbone component of the local task gradient is


$$\begin{array}{c} q_{i}=\nabla_{\theta }{ℒ}_{i}. \end{array}$$ (14)

The vector $q_{i}$ is stored in ${ℬ}_{i}$ after the local optimizer step. At synchronization, Eq 8 converts the recent records into one interval-mean gradient per task, and GFT directly uses the stored gradients as recent first-order summaries for the additional coordination correction. The local update preserves task-wise adaptation, whereas the periodic branch adjusts the shared backbone using information collected across tasks. A scheduled synchronization is executed only after every task has contributed at least one gradient to the current records; otherwise, the records are retained while local sampling continues.

For the fusion-ratio sensitivity study, we manually define λ∈[0,1] as the strength of this periodic coordination correction:


$$\begin{array}{c} \theta \leftarrow \theta -\eta \lambda \tilde{g}. \end{array}$$ (15)

The learned task weights $\alpha_{i}$ and the fusion ratio λ operate at different levels. Specifically, $\alpha_{i}$ determines the relative contribution of task *i*’s interval-mean gradient to the fused direction $\tilde{g}$, whereas λ scales the overall strength of the periodic coordination correction applied to the shared backbone. The main HeteroGrad-DT model uses λ=1. For 0<λ≤1, λ scales only the shared-backbone coordination correction, while GFT is trained with its learning rate β. Setting λ=0 skips the GFT forward pass, the coordination correction, and the GFT parameter update, which exactly defines the w/o GFT configuration. The manually specified λ=0.5 setting therefore applies a half-strength coordination correction without changing the ordinary HAP local updates.

The GFT architecture and the flow from task descriptors to the fused gradient are illustrated in Fig 3.

**Fig 3:**
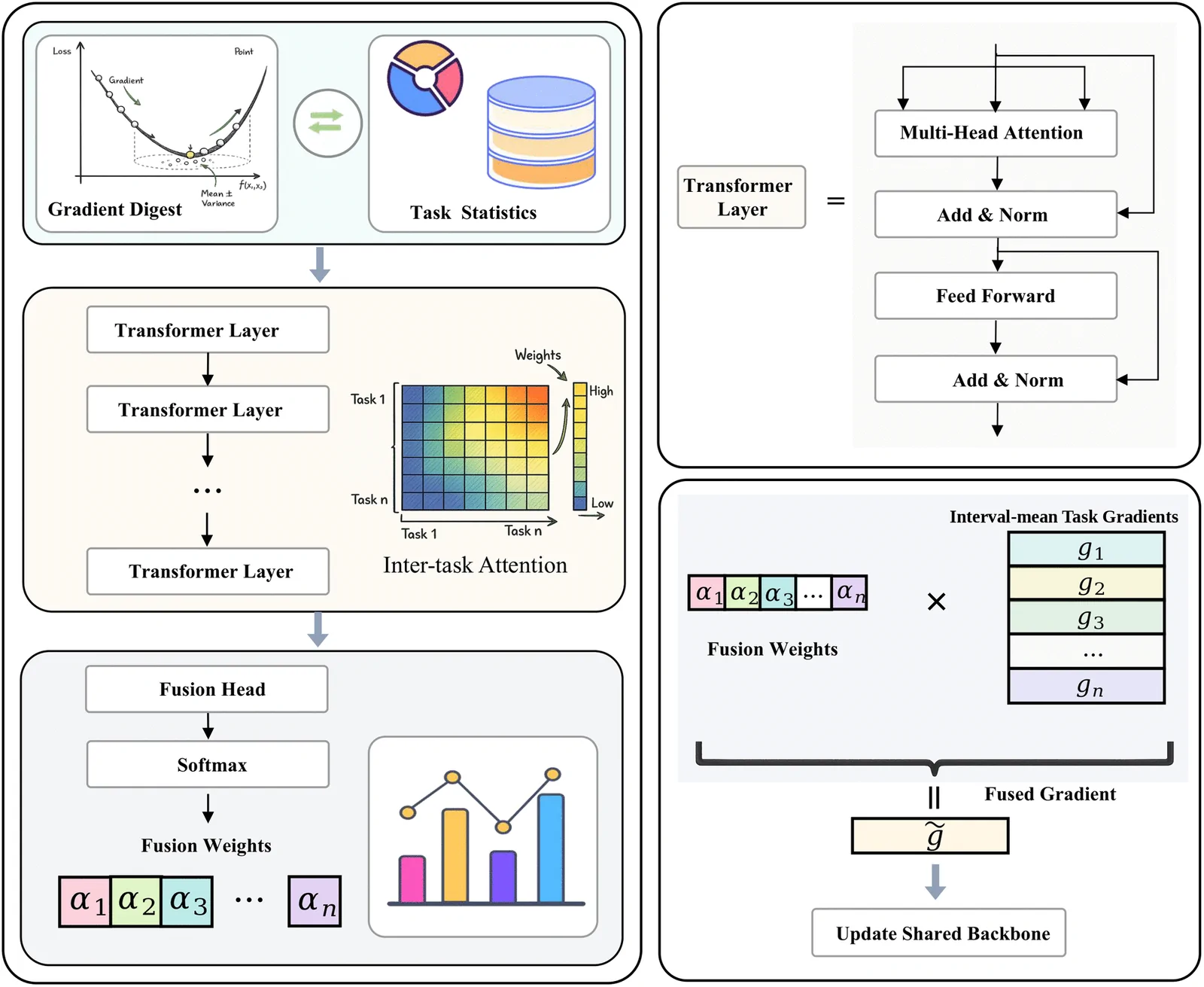
Architecture of the Gradient Flow Transformer. GFT computes adaptive fusion weights from Task Statistics and Gradient Digest features through self-attention. These weights combine the interval-mean task gradients $g_{i}$ into a fused gradient for updating the shared backbone. The encoder stack is shown schematically; the main experiments use two GFT encoder layers with four attention heads.

**Algorithm 1. Training procedure of HeteroGrad-DT**

**Require:** Training tasks ${𝒯}_{train}$, datasets $\{{𝒟}_{i}{\}}_{i=1}^{N}$, total iterations *E*, synchronization interval *E*_sync_, learning rates η and β, manually set fusion ratio λ∈[0,1]

**Ensure:** Trained shared backbone and HAP interfaces and, when λ>0, GFT parameters ϕ *Initialization*

1  **for each** task ${𝒯}_{i}\in {𝒯}_{train}$ **do**

2   Compute fixed-dimensional Task Statistics $f_{stat}^{\left(i\right)}$ from ${𝒟}_{i}$

3   Initialize interval gradient record ${ℬ}_{i}\leftarrow \emptyset$

4  **end for**

*Ordinary HAP updates and periodic coordination*

5  **for** *t* = 1 to *E* **do**

6   Sample task ${𝒯}_{i}$ uniformly and batch $\tau_{i}$ uniformly from ${𝒟}_{i}$

7   Construct RTG, state, and action tokens with the HAP interfaces for ${𝒯}_{i}$

8   Predict actions with the shared backbone and task-specific head $h_{i}$

9   Compute ${ℒ}_{i}$ and let $q_{i}\leftarrow \nabla_{\theta }{ℒ}_{i}$ be its shared-backbone gradient

10  Apply the ordinary HAP optimizer update for the sampled task

11   Append $q_{i}$ to ${ℬ}_{i}$

12   **if** $t\equiv 0\;(\mathrm{mod}\;E_{sync})$ and $\begin{array}{c} B_{j}=\;\;\;\;\;/\emptyset \end{array}$ for every task *j* **then**

13    **if** λ>0 **then**

*GFT synchronization*

14     **for each** task $\begin{array}{c} {𝒯}_{j}\in {𝒯}_{train} \end{array}$ **do**

15      $g_{j}\leftarrow |{ℬ}_{j}{|}^{-1}\sum_{q\in {ℬ}_{j}}q$

16      Compute $f_{grad}^{\left(j\right)}$ using Eq 9

17      $f_{j}\leftarrow [f_{stat}^{\left(j\right)}∥f_{grad}^{\left(j\right)}]$

18     **end for**

19     $\alpha \leftarrow GFT(\{f_{j}{\}}_{j=1}^{N};\phi )$

20     $\tilde{g}\leftarrow \sum_{j=1}^{N}\alpha_{j}g_{j}$

21     $\theta \leftarrow \theta -\eta \lambda \tilde{g}$        *(periodic coordination correction)*

22     $\phi \leftarrow \phi -\beta \nabla_{\phi }{ℒ}_{SPM}$

23    **end if**

24    Refresh all interval gradient records ${ℬ}_{j}\leftarrow \emptyset$

25   **end if**

26  **end for**

## Experiments

### Task datasets and experimental setup

**Benchmarks and data.** We use the DeepMind Control Suite (DMControl) [31]. Following the task collection used by TD-MPC2 [32], the largest benchmark contains 30 tasks comprising 19 standard environments and 11 custom extensions. The complete MT30 task list and the corresponding observation and action dimensions are provided in Table 1. The MT5 and MT10 composition and ordering are reported in the note to Table 1. We use the medium-replay datasets with 2,000 trajectories per task. The same per-task trajectories and data partitions are used for all compared methods. Following the TD-MPC2 DMControl protocol, we report $S_{i}=100{\bar{R}}_{i}/1000$ for task *i* and average $S_{i}$ uniformly across tasks, where ${\bar{R}}_{i}$ is the mean undiscounted episodic return [31,32].

**Table 1:** Detailed specifications of the 30 DeepMind Control Suite tasks used in the experiments.

Task Name	Obs.	Act.	Task Name	Obs.	Act.
Acrobot Swingup	6	1	Fish Swim	24	5
Cartpole Balance	5	1	Hopper Hop	15	4
Cartpole Balance Sparse	5	1	Hopper Hop Backwards	15	4
Cartpole Swingup	5	1	Hopper Stand	15	4
Cartpole Swingup Sparse	5	1	Pendulum Spin	3	1
Cheetah Jump	17	6	Pendulum Swingup	3	1
Cheetah Run	17	6	Reacher Easy	6	2
Cheetah Run Back	17	6	Reacher Hard	6	2
Cheetah Run Backwards	17	6	Reacher Three Easy	8	3
Cheetah Run Front	17	6	Reacher Three Hard	8	3
Cup Catch	8	2	Walker Run	24	6
Cup Spin	8	2	Walker Run Backwards	24	6
Finger Spin	9	2	Walker Stand	24	6
Finger Turn Easy	12	2	Walker Walk	24	6
Finger Turn Hard	12	2	Walker Walk Backwards	24	6

Observation dimensions range from 3 to 24 and action dimensions from 1 to 6.

*Subset order.* MT5: Cartpole Balance, Cartpole Swingup, Cup Catch, Pendulum Spin, Reacher Easy. MT10: Walker Stand, Cheetah Run, Cartpole Balance, Cartpole Swingup Sparse, Cup Catch, Finger Spin, Hopper Hop, Walker Walk Backwards, Cup Spin, Pendulum Spin.

**Evaluation protocol.** Main performance results are reported over five random seeds after 1M training iterations. Each evaluation checkpoint uses 10 episodes per task. All methods use matched task sets, offline trajectories, evaluation checkpoints, and seed assignments. The training-cost experiment uses the same 1M-iteration budget and reports its hardware separately in the training-cost analysis.

**Implementation details.** The main shared-backbone, optimization, and GFT hyperparameters are summarized in Table 2.

**Table 2:** Hyperparameters used for HeteroGrad-DT in the main experiments.

Parameter	Value
Number of Transformer layers	6
Number of attention heads	8
Embedding dimension *d*	256
Context length *K*	20
Prompt length $K_{p}$	5
Dropout	0.1
Batch size	16
Optimizer	Adam
Ordinary HAP/backbone learning rate η	$3.0\times 10^{-5}$
Weight decay	$1.0\times 10^{-4}$
Gradient clipping	0.25
RTG scale	100
Maximum training iterations	$1.0\times 10^{6}$
Evaluation episodes per task	10
GFT synchronization interval *E*_sync_	5000
GFT hidden dimension	64
GFT encoder layers/attention heads	2/4
GFT learning rate β	$1.0\times 10^{-4}$
Fusion ratio λ	1.0

**Baselines.** We compare HeteroGrad-DT with representative methods covering shared-backbone training, prompt conditioning, parameter-space masking, gradient projection, and static grouping:

- **MTDT**: Multi-Task Decision Transformer with direct parameter sharing.
- **Prompt-DT**: A trajectory-prompted method that uses in-context information for task adaptation.
- **HarmoDT**: A subspace-based method that learns task-specific masks to mitigate interference.
- **HAP + PCGrad**: A geometric projection method applied on top of the HAP interface.
- **Prompt-DT-G**: A static grouping method applied on top of the HAP interface. Tasks are grouped once before training using fixed dataset-level structural statistics, including state/action dimensions and kinematic summary statistics, and the assignments remain fixed throughout training.

### Main results

All results in Table 3 are evaluated over five random seeds. We report mean ± standard deviation together with 95% confidence intervals. For mean $\bar{x}$, sample standard deviation *s*, and *n* = 5 seeds, the interval is $\bar{x}\pm t_{0.975,4}s/\sqrt{5}$, where *t*_0.975,4_ = 2.776. Confidence intervals are computed from the unrounded seed-level values; the displayed means and standard deviations are rounded only after the interval calculation.

**Table 3:** Averaged normalized scores with standard deviations and 95% confidence intervals over five seeds.

Method	5 Tasks	10 Tasks	30 Tasks
MTDT	72.36±1.76 [70.17, 74.54]	69.72±1.66 [67.66, 71.78]	32.05±2.33 [29.16, 34.94]
Prompt-DT	76.22±1.14 [74.80, 77.64]	71.88±2.05 [69.33, 74.42]	39.53±2.72 [36.16, 42.91]
HarmoDT	73.07±1.42 [71.30, 74.84]	70.31±1.78 [68.10, 72.52]	31.74±1.63 [29.71, 33.77]
HAP + PCGrad	78.28±1.55 [76.36, 80.21]	73.24±1.64 [71.20, 75.27]	39.05±1.29 [37.45, 40.65]
Prompt-DT-G	75.52±1.62 [73.51, 77.53]	77.16±1.20 [75.67, 78.65]	36.30±1.94 [33.89, 38.71]
**HeteroGrad-DT**	83.46±1.18 [82.00, 84.92]	77.99±1.16 [76.55, 79.43]	42.40±1.23 [40.87, 43.93]

Values are reported as mean ± standard deviation [95% confidence interval]. Confidence intervals are computed from unrounded seed-level statistics. The best result in each setting is highlighted in bold.

**Performance across task scales.** HeteroGrad-DT achieves the highest mean score across the 5-, 10-, and 30-task settings. On MT30, it reaches 42.40, exceeding MTDT by 10.35 points, or approximately 32.3%. The result is consistent with a benefit from task-specific interfaces and periodic gradient coordination in the larger heterogeneous task set.

**Comparison with geometric projection.** HeteroGrad-DT exceeds HAP + PCGrad by 3.35 points on MT30, corresponding to approximately 8.6%. HAP + PCGrad applies stepwise geometric projection to the shared-backbone gradients, whereas HeteroGrad-DT accumulates task gradients within each synchronization interval before applying the learned fused correction. This difference preserves local adaptation between synchronization steps while still controlling destructive cross-task interference.

**Comparison with parameter masking and task grouping.** HeteroGrad-DT also outperforms HarmoDT and Prompt-DT-G. HarmoDT manages interference through task-specific parameter masks, while Prompt-DT-G relies on a fixed grouping of tasks. HeteroGrad-DT achieves higher mean scores than the parameter-masking and fixed-grouping baselines in the evaluated settings.

Taken together, the main results indicate higher mean performance across all three task scales, with the clearest relative gain observed over direct shared-backbone training on MT30.

### Ablation and mechanism analysis

**Component-removal ablation.** Table 4 uses the complete HeteroGrad-DT model as the reference and removes one component at a time. Removing GFT lowers the scores from 83.46/77.99/42.40 to 80.35/75.24/40.69, supporting a contribution from the GFT coordination branch beyond the task-aware interface. Removing HAP lowers the scores to 79.68/74.62/40.58, which is consistent with a beneficial role of task-specific tokenizers and prediction heads when GFT is retained. The two components therefore address complementary sources of difficulty: HAP handles task-dependent input and output dimensionality at the model interfaces, whereas GFT coordinates the shared update.

**Table 4:** Component-removal ablation of HeteroGrad-DT on the 5-, 10-, and 30-task DMControl benchmarks.

Variant	5 Tasks	10 Tasks	30 Tasks
**Full HeteroGrad-DT**	83.46±1.18	77.99±1.16	42.40±1.23
w/o GFT	80.35±1.37	75.24±1.52	40.69±1.70
w/o HAP	79.68±1.48	74.62±1.61	40.58±1.68

Results are mean ± standard deviation over five seeds.

**HAP compatibility across backbones.** Table 5 applies the HAP interface to Prompt-DT and HarmoDT. Prompt-DT + HAP is the same architecture and training configuration as the w/o GFT row in Table 4; the same five-seed statistics are therefore reported in both tables. The higher mean scores obtained after adding HAP to both backbones suggest that the task-aware interface is compatible with different multi-task optimization strategies.

**Table 5:** Effect of HAP across different backbone variants.

Method/Variant	5 Tasks	10 Tasks	30 Tasks
Prompt-DT	76.22±1.14	71.88±2.05	39.53±2.72
Prompt-DT + HAP	80.35±1.37	75.24±1.52	40.69±1.70
HarmoDT	73.07±1.42	70.31±1.78	31.74±1.63
HarmoDT + HAP	78.26±1.33	74.08±1.55	34.86±1.72

Results are mean ± standard deviation over five seeds. Prompt-DT + HAP uses the same architecture and training configuration as the w/o GFT variant in Table 4; therefore, the same five-seed statistics are reported.

We examine the action-level effect of the interface design on the fixed MT10 validation split. In Fig 4, T1–T10 follow the MT10 task order reported in the note to Table 1. The unified padding interface zero-pads task-native state/action vectors to common dimensions and uses a shared prediction interface, whereas HAP uses task-specific projections and prediction heads. Both settings use the same shared sequence backbone and the same held-out trajectories. For $M_{i}$ target action scalars from task *i*, validation MSE and absolute mean error are


$$\begin{array}{c} {MSE}_{i}=\frac{1}{M_{i}}\sum_{m=1}^{M_{i}}({\hat{a}}_{m}-a_{m}{)}^{2},\;{AME}_{i}=\left|\frac{1}{M_{i}}\sum_{m=1}^{M_{i}}({\hat{a}}_{m}-a_{m})\right|. \end{array}$$ (16)

**Fig 4:**
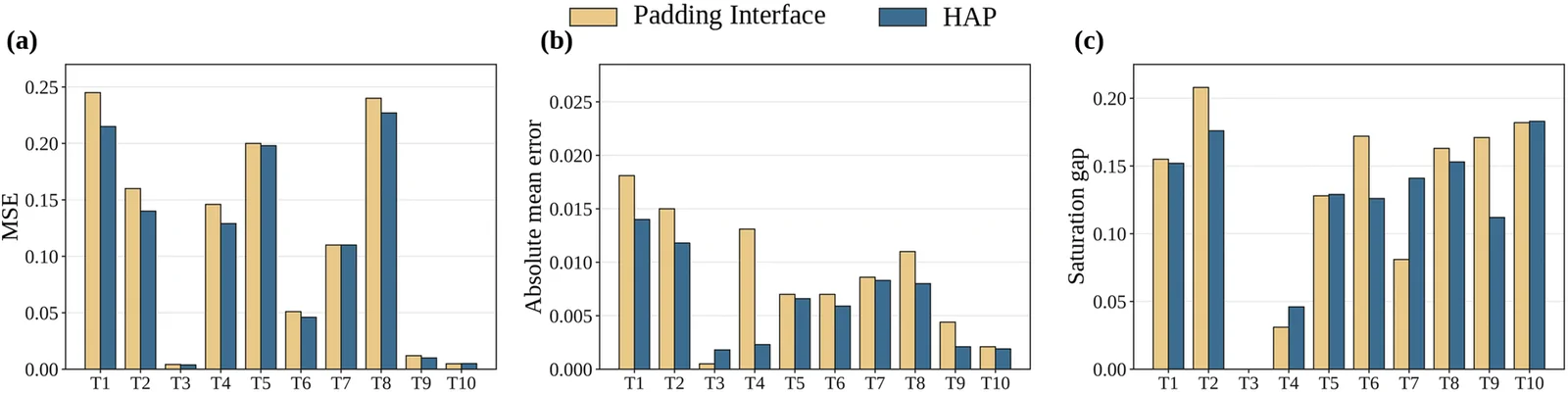
Action-prediction analysis of HAP versus a unified padding interface on the fixed MT10 validation split. T1–T10 follow the MT10 task order reported in the note to Table 1. The panels report the metrics defined in Eqs 16 and 17; lower values indicate closer matching to held-out target actions.

The saturation gap compares predicted and target boundary-action frequencies using a fixed threshold ρ for actions normalized to [−1,1]:


$$\begin{array}{c} {SatGap}_{i}=\left|\frac{1}{M_{i}}\sum_{m=1}^{M_{i}}𝕀(|{\hat{a}}_{m}|\geq \rho )-\frac{1}{M_{i}}\sum_{m=1}^{M_{i}}𝕀(|a_{m}|\geq \rho )\right|. \end{array}$$ (17)

HAP generally lowers the MSE and absolute mean error, which is consistent with lower squared prediction error and smaller aggregate action bias under task-specific output interfaces. The saturation-gap results do not show uniform improvement across tasks.

**Gradient coordination.** Table 6 reports the conflict rate and coordination score over five seeds under the matched MT30 gradient-diagnostic protocol. The conflict rate is the fraction of task-gradient pairs with negative cosine similarity, whereas the coordination score is the average alignment between each task gradient and the final shared update direction. For each seed, diagnostics are computed offline at the same saved MT30 checkpoints for all compared methods. At each checkpoint, one batch from each task is used to compute shared-backbone task gradients; conflict rate is averaged over all task-gradient pairs, and coordination score is averaged over all tasks after forming the final shared update direction. Checkpoint-level values are then averaged within each seed, and Table 6 reports mean ± standard deviation across the five seeds. Compared with the model without GFT, HeteroGrad-DT reduces the conflict rate from 0.4000 to 0.2667 and increases the coordination score from 0.3193 to 0.3311. HarmoDT obtains a similar coordination score but retains a higher conflict rate. These metrics describe different properties: positive average alignment with the final shared update may coexist with negative pairwise interactions among tasks. In this diagnostic, the main observed difference is the lower rate of negative pairwise interactions while positive average alignment with the final shared update is maintained.

**Table 6:** Gradient-conflict and coordination metrics over five seeds under the matched MT30 diagnostic protocol, computed offline for mechanism analysis.

Method	Conflict rate	Coordination score
HeteroGrad-DT	0.2667±0.0196	0.3311±0.0124
Without GFT	0.4000±0.0261	0.3193±0.0152
HarmoDT	0.3598±0.0227	0.3351±0.0131

Results are mean ± standard deviation.

**Descriptor-input ablation.** Table 7 compares the No GFT, Gradient Digest only, Task Statistics only, and Task Statistics + Gradient Digest settings while keeping the remaining training settings unchanged; the No GFT row repeats the w/o GFT control in Table 4. The combined descriptor gives the highest mean across all three task scales, supporting the use of both task-level statistics and interval-level gradient information.

**Table 7:** Ablation of GFT descriptor inputs on the 5-, 10-, and 30-task benchmarks.

GFT descriptor input	5 Tasks	10 Tasks	30 Tasks
No GFT	80.35±1.37	75.24±1.52	40.69±1.70
Gradient Digest only	80.93±1.40	75.65±1.48	40.87±1.61
Task Statistics only	82.08±1.29	76.58±1.34	41.64±1.43
Task Statistics + Gradient Digest	83.46±1.18	77.99±1.16	42.40±1.23

Results are mean ± standard deviation over five seeds. The No GFT row is identical to the w/o GFT configuration in Table 4.

### GFT hyperparameter sensitivity

We evaluate four GFT hyperparameters: learning rate, hidden dimension, fusion ratio, and synchronization interval, with mean performance and standard-deviation bands computed over five seeds. The default configuration uses a GFT learning rate of 1e−4, hidden dimension 64, fusion ratio λ=1.0, and a synchronization interval of 5000 training iterations. These settings reproduce the HeteroGrad-DT results in Table 3, namely 83.46, 77.99, and 42.40 on the 5-, 10-, and 30-task benchmarks. In the manually defined fusion-ratio sweep, λ∈{0,0.5,1} controls the strength of the GFT synchronization branch according to Eq 15: λ=0 matches w/o GFT, λ=0.5 is the intermediate half-strength setting, and λ=1 uses full learned GFT fusion.

The learning-rate sweep shows that 1e−4 gives the strongest results, while larger learning rates reduce performance across the three task scales. A hidden dimension of 64 provides the best overall balance between capacity and stability. For the fusion-ratio comparison, performance improves toward full learned fusion, although the response differs across task scales. On MT5, λ=0.5 provides a moderate gain over λ=0, followed by a larger improvement at λ=1. On MT10, the change at λ=0.5 is relatively small, whereas full fusion produces a clearer improvement. On MT30, performance increases more steadily as λ increases from 0 to 1. Finally, a synchronization interval of 5000 gives the best overall result. The 10000 setting remains close on 5 tasks but is lower on the 10- and 30-task benchmarks, indicating that an intermediate synchronization interval is more stable across task scales.

The complete sensitivity results are summarized in Fig 5.

**Fig 5:**
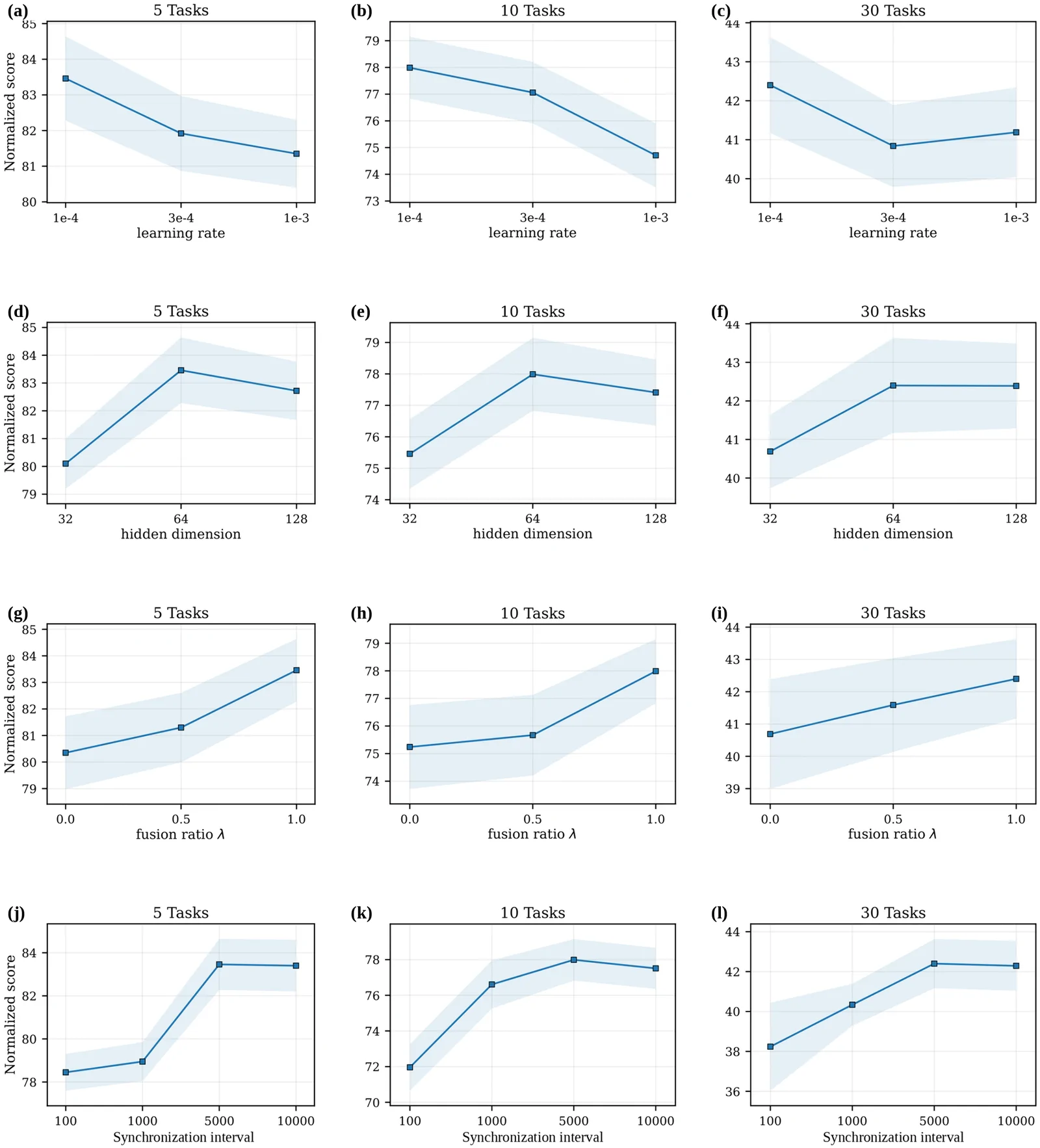
Sensitivity analysis of GFT hyperparameters on the 5-, 10-, and 30-task benchmarks. The evaluated factors are GFT learning rate, hidden dimension, fusion ratio, and synchronization interval. The fusion ratio is manually set to λ∈{0,0.5,1}, where λ=0 matches the w/o GFT configuration in Table 4, λ=0.5 applies half-strength GFT synchronization, and λ=1 denotes full learned GFT fusion. The synchronization interval denotes the number of training iterations between synchronization steps, so a larger value corresponds to less frequent synchronization. Shaded regions denote the standard deviation across five seeds. The best/default settings match the HeteroGrad-DT results reported in Table 3.

### Training cost and memory usage

Table 8 reports a matched MT30 comparison for 1M training iterations on a single NVIDIA RTX 4090 GPU. HeteroGrad-DT has more parameters and higher peak memory than Prompt-DT because it adds task-specific interfaces and GFT-related states. However, the additional capacity does not lead to a proportional slowdown. The shared causal Transformer still dominates per-iteration computation, the task-specific interfaces are lightweight, and GFT is invoked only at synchronization steps. As a result, HeteroGrad-DT remains close to Prompt-DT in both throughput and training time.

**Table 8:** Matched MT30 training cost benchmark for 1M training iterations.

Method	Params	Throughput (iter/s)	Training time (h)	Peak memory (MiB)
HeteroGrad-DT	7.36M	43.54	6.38	11928
HarmoDT	5.29M	21.20	13.10	10999
Prompt-DT	5.29M	43.00	6.46	6529

All methods use the same dataset, batch size, context length, prompt length, and runtime environment. Training time is measured over the complete 1M-iteration run and excludes evaluation rollouts.

HarmoDT is slower in our implementation because mask and subspace operations participate in regular backbone updates and add parameter-selection and masking overhead. Overall, HeteroGrad-DT mainly trades additional memory for task-aware interfaces and gradient coordination while preserving training throughput close to the shared-backbone baseline.

## Discussion

The results support a two-part interpretation of HeteroGrad-DT. HAP addresses structural mismatch at the input and output interfaces, whereas GFT addresses interference within the shared optimization process. In the component-removal study, removing either module lowers the mean performance across all three task scales, supporting complementary rather than interchangeable roles. The particularly clear relative gain over direct shared-backbone training on MT30 further suggests that explicit interface design and learned update coordination become useful when a single sequence model must absorb increasingly diverse task structures.

The HAP analyses indicate that heterogeneous interfaces directly affect how the shared model allocates its representation capacity. When all tasks are forced through a padded input and output interface, the backbone must simultaneously learn temporal regularities and compensate for task-dependent dimensional and semantic mismatches. The consistent gains obtained by adding HAP to both Prompt-DT and HarmoDT suggest that task-specific tokenizers and prediction heads reduce this avoidable burden independently of the particular optimization strategy used by the backbone. The action-prediction analysis supports this interpretation: lower validation MSE and absolute mean error are consistent with lower squared prediction error and smaller aggregate action bias under the HAP interfaces, whereas the saturation-gap results do not show uniform improvement across tasks. Taken together, these results suggest that HAP improves multi-task learning by resolving structural differences at the model boundary, allowing the shared Transformer to focus more directly on temporal structure shared across tasks.

The GFT analyses provide complementary evidence at the optimization level. The lower pairwise conflict rate, together with positive alignment between the task gradients and the final shared update, is consistent with periodic coordination complementing rather than replacing ordinary task-wise learning. Local HAP updates retain task-specific adaptation, while GFT uses the recent gradient records to reduce directions that are poorly compatible with the wider task set when forming the shared correction. This interpretation is also consistent with the larger relative gain observed on MT30, where a single backbone must accommodate a broader range of state–action structures and competing update directions. The sensitivity results further suggest that coordination requires a balance between local adaptation and cross-task correction. Very frequent synchronization provides only short local intervals, whereas overly infrequent synchronization aggregates older optimization signals; the intermediate interval performs best across the three task scales. Because GFT is invoked periodically rather than at every local update, this additional coordination preserves throughput close to Prompt-DT, although it requires extra memory for task-specific interfaces and gradient records.

The current evidence is limited to state-based simulated control, and performance under physical robot deployment remains to be validated. Real systems introduce sensing noise, actuation delay, hardware constraints, dynamics mismatch, and safety requirements that are not represented by the current DMControl evaluation. The largest benchmark also contains 30 tasks, so the behavior of the shared backbone and the GFT descriptor as task collections grow substantially larger remains to be examined. In addition, the fixed-dimensional Task Statistics and interval-mean Gradient Digest deliberately compress rich task and optimization information; more complex or rapidly changing task relations may require more expressive descriptors or adaptive synchronization. Future work will evaluate HeteroGrad-DT on physical robots or higher-fidelity sim-to-real benchmarks, extend the framework to visual observations, and study adaptive descriptor construction and synchronization policies for larger and more diverse task collections.

## Conclusion

This paper presented HeteroGrad-DT for heterogeneous multi-task offline reinforcement learning. The method addresses two coupled challenges: state-action interface mismatch and cross-task optimization interference. HAP provides task-specific input and output interfaces around a shared Decision Transformer backbone, while GFT periodically coordinates task gradients using Task Statistics and Gradient Digest features.

Experiments on the DeepMind Control Suite show that HeteroGrad-DT improves average normalized return over representative multi-task methods across all three task scales, with a particularly clear relative gain over direct shared-backbone training on MT30. The component-removal results support complementary roles for HAP and GFT. Further analyses cover HAP compatibility across backbones, action prediction, pairwise gradient conflict, GFT hyperparameter sensitivity, and training cost. Together, these results support the benefit of coupling task-aware representation interfaces with optimization-space coordination in the evaluated heterogeneous multi-task offline learning settings.

The main evaluation remains limited to simulated control and a maximum of 30 tasks. Future work will examine larger task collections, richer observation modalities, and more realistic robotic deployment conditions.
